# DPTIP, a newly identified potent brain penetrant neutral sphingomyelinase 2 inhibitor, regulates astrocyte-peripheral immune communication following brain inflammation

**DOI:** 10.1038/s41598-018-36144-2

**Published:** 2018-12-07

**Authors:** Camilo Rojas, Elena Barnaeva, Ajit G. Thomas, Xin Hu, Noel Southall, Juan Marugan, Amrita Datta Chaudhuri, Seung-Wan Yoo, Niyada Hin, Ondrej Stepanek, Ying Wu, Sarah C. Zimmermann, Alexandra G. Gadiano, Takashi Tsukamoto, Rana Rais, Norman Haughey, Marc Ferrer, Barbara S. Slusher

**Affiliations:** 1 Johns Hopkins Drug Discovery, Johns Hopkins School of Medicine, Baltimore, Maryland 21205 USA; 2 Department of Molecular and Comparative Pathobiology, Johns Hopkins School of Medicine, Baltimore, Maryland 21205 USA; 3 Department of Neurology, Johns Hopkins School of Medicine, Baltimore, Maryland 21205 USA; 4 Department of Psychiatry and Behavioral Sciences, Johns Hopkins School of Medicine, Baltimore, Maryland 21205 USA; 5 Department of Neuroscience, Johns Hopkins School of Medicine, Baltimore, Maryland 21205 USA; 6 Department of Medicine, Johns Hopkins School of Medicine, Baltimore, Maryland 21205 USA; 7 Department of Oncology, Johns Hopkins School of Medicine, Baltimore, Maryland 21205 USA; 8 National Center for Advancing Translational Sciences (NCATS), National Institute of Health, Bethesda, MD 20892-3370 USA

## Abstract

Brain injury and inflammation induces a local release of extracellular vesicles (EVs) from astrocytes carrying proteins, RNAs, and microRNAs into the circulation. When these vesicles reach the liver, they stimulate the secretion of cytokines that mobilize peripheral immune cell infiltration into the brain, which can cause secondary tissue damage and impair recovery. Recent studies suggest that suppression of EV biosynthesis through neutral sphingomyelinase 2 (nSMase2) inhibition may represent a new therapeutic strategy. Unfortunately, currently available nSMase2 inhibitors exhibit low potency (IC_50_ ≥ 1 μM), poor solubility and/or limited brain penetration. Through a high throughput screening campaign of >365,000 compounds against human nSMase2 we identified 2,6-Dimethoxy-4-(5-Phenyl-4-Thiophen-2-yl-1H-Imidazol-2-yl)-Phenol (DPTIP), a potent (IC_50_ 30 nM), selective, metabolically stable, and brain penetrable (AUC_brain_/AUC_plasma_ = 0.26) nSMase2 inhibitor. DPTIP dose-dependently inhibited EV release in primary astrocyte cultures. In a mouse model of brain injury conducted in GFAP-GFP mice, DPTIP potently (10 mg/kg IP) inhibited IL-1β-induced astrocyte-derived EV release (51 ± 13%; p < 0.001). This inhibition led to a reduction of cytokine upregulation in liver and attenuation of the infiltration of immune cells into the brain (80 ± 23%; p < 0.01). A structurally similar but inactive analog had no effect *in vitro* or *in vivo*.

## Introduction

Neutral sphinghomyelinase 2 (nSMase2) catalyzes the formation of ceramide, a required step in the formation and release of extracellular vesicles (EVs)^1^. EVs are involved in intercellular communication underlying many physiological and pathological processes^2–4^. While transient increases in nSMase2 activity are part of normal physiological function, chronic inflammation, largely through TNFα and IL-1β signaling, is known to upregulate nSMase2^5^. Upregulation of nSMase2 activity is associated with cognitive impairment in HIV infection^6^, and with plaque deposition in AD^7,8^. Moreover, astrocyte-derived EVs (ADEVs) isolated from the plasma of AD patients contain increased amounts of complement proteins, implying that glial activation leads to the release of EVs that may play some role in regulating innate immunity^9^. Our group has shown that brain inflammation, a common theme in many neurodegenerative disorders^10^, can trigger the release of EVs from astrocytes which primes the infiltration of immune cells into the brain via upregulation of cytokines in the periphery^11^. Taken together, inhibition of EV secretion through inhibition of nSMAse2 is emerging as a novel avenue for the treatment of diseases associated with aberrant exosomal intercellular communication^11–13^. Unfortunately, limitations of currently available nSMase2 inhibitors have prevented a detailed evaluation of the role of nSMase2 in disease models and the advancement of drug-like nSMase2 inhibitors to the clinic. Currently available nSMase2 inhibitors have low potency (IC_50_’s in µM level), poor aqueous solubility, and/or limited brain penetration. GW4869^14^, the most widely used inhibitor, has low inhibitory potency (IC_50_ = 1 µM) in biochemical assays and very poor solubility (practically insoluble in water with poor solubility in organic solvents such as DMSO (0.2 mg/ml). These attributes have hampered GW4869′s clinical development. Cambinol, an inhibitor our group identified from a pilot screen of commercially available small chemical libraries^15^ showed better solubility, but it was metabolically unstable and exhibited a poor *in vivo* pharmacokinetic profile. Chemistry efforts by our laboratory to improve cambinol’s potency (IC_50_ = 5 µM) and stability were unsuccessful. Herein, we report on a high throughput screening (HTS) campaign of over 365,000 compounds that identified a potent inhibitor of nSMase2 termed DPTIP, with an excellent pharmacokinetic profile including significant brain penetration, which was capable of dose-dependently blocking EV release from primary astrocytes. Moreover, in a mouse model of brain inflammation that recapitulates common features of neurodegenerative diseases, DPTIP potently inhibited IL-1β-induced ADEV release, peripheral cytokine upregulation and neutrophil migration into the brain.

## Results and Discussion

### Development of a 1536-well cell-free human recombinant nSMase2 enzyme activity assay

**Figure 1:**
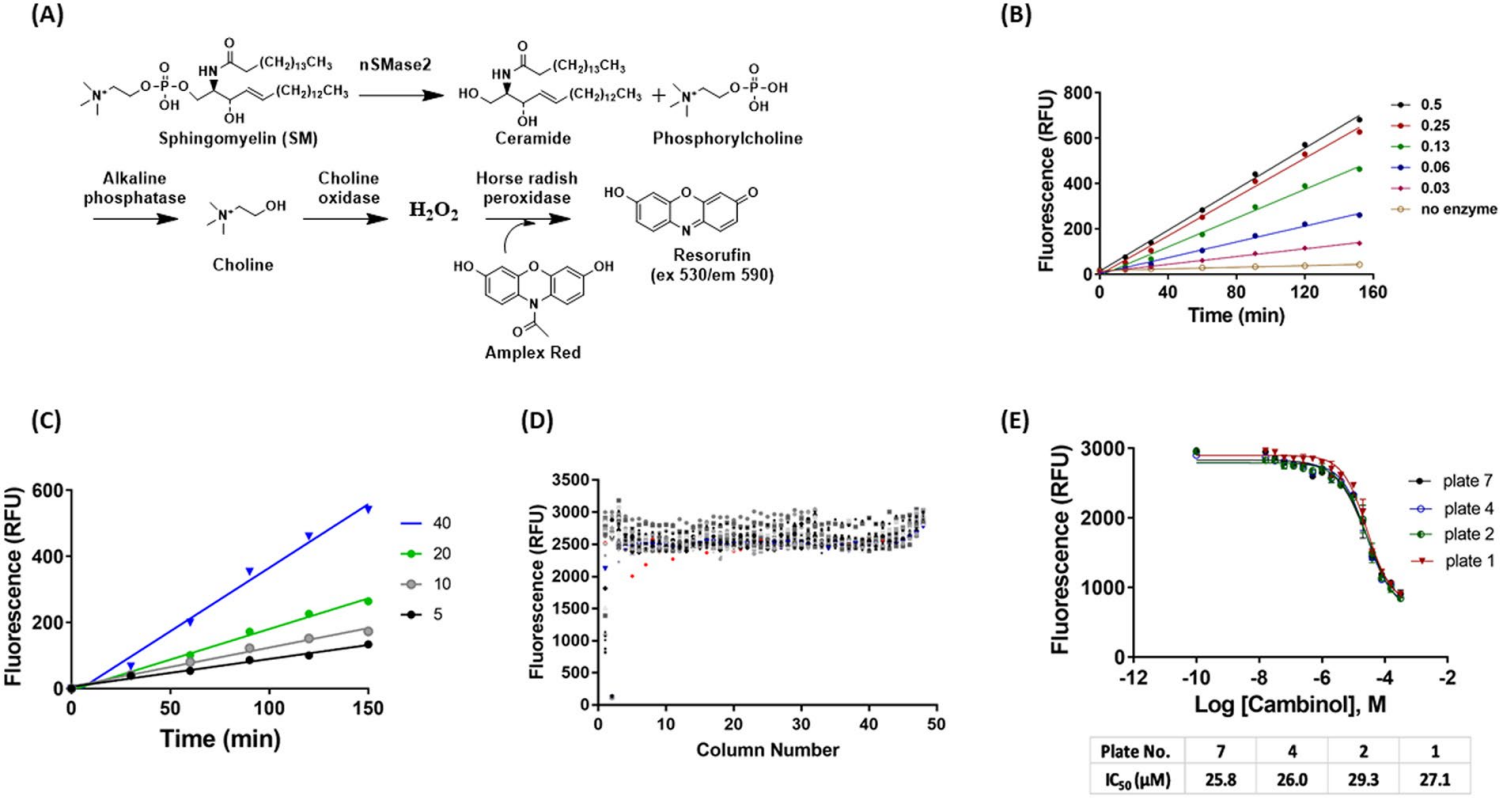
Validation of the human nSMase2 fluorescence-based assay in 1536-well format to screen for inhibitors of the enzyme in dose response quantitative HTS. (**A**) Schematic representation of the assay - Human nSMase2 catalyzes the hydrolysis of sphingomyelin (SM) to ceramide and phosphorylcholine. Using alkaline phosphatase, choline oxidase, horse radish peroxidase and Amplex Red, phosphorylcholine is stoichiometrically converted through enzyme-coupled reactions to fluorescent resorufin; fluorescence is directly proportional to nSMase2 activity. (**B**) Dependence of fluorescence signal on time of incubation (in min) at several enzyme concentrations (0.03 to 0.5 µg protein/µL) in the presence of 20 µM SM. (**C**) Dependence of fluorescence signal on time of incubation at different SM concentrations (0.005 to 0.04 mM) in the presence of 0.063 µg protein/µL. (**D**) Scatter plot of fluorescence signal from a 1536-well assay plate. - Human nSMase2 cell lysate (0.1 µg/µL) was incubated with SM (20 µM) and coupling reagents for 2 h at 37 °C before measuring fluorescence. When using cambinol as positive control, compound was preincubated with human nSMase2 for 15 min. Column 1: Cambinol dose response. Column 2: Negative control (no enzyme). Column 3: Positive control (bacterial SMase 0.02 U/mL). Columns 4–48 human nSMase2 (Final DMSO concentration: 0.57%). Fluorescence signal is expressed as relative fluorescent units (RFU) on the y-axis. Plate number is shown on x-axis. (**E**) Dose response of inhibition of nSMase2 by cambinol, a known inhibitor of nSMase2^15^. – Wells contained cell lysate prepared from cells expressing nSMase2 (0.1 µg/µL) and SM (20 µM) with increasing concentrations of cambinol as indicated. During the screen, cambinol was used to track plate-to-plate variability; it was delivered onto each plate in 16 doses, at 1:2 dilutions in the range 285 µM – 17 nM.

Human nSMase2 catalyzes the hydrolysis of sphingomyelin (SM) to phosphorylcholine and ceramide. As we reported previously, we used the Amplex Red system to monitor nSMase2 activity^15^. In this reaction, one of the enzymatic products, phosphorylcholine, is stoichiometrically converted through a series of enzyme-coupled reactions to fluorescent resorufin, so that fluorescence signal is directly proportional to nSMase2 activity (Fig. 1A). An enzymatic assay protocol was developed in 1536-well format for implementation for HTS. Several parameters were first optimized through the measurement of the fluorescence signal. Fluorescence signal increased with longer times of incubation (15–150 min) and increasing nSMase2 concentrations (0.03 to 0.5 µg protein/mL) at a constant SM concentration (20 µM) (Fig. 1B). Similarly, fluorescence signal increased with longer time of incubation (30–150 min) and increasing SM concentrations (5–40 µM) at a constant enzyme concentration (0.063 µg protein/ml) (Fig. 1C). Based on these results, we chose 0.1 µg protein/mL human nSMase2 cell lysate, 20 µM SM in a total volume of 4 µL and 2 h incubation at 37 °C to assess assay performance in HTS format. Under these conditions, reaction rate was linear with a robust fluorescence signal of approximately 2500 relative fluorescent units (RFU). Cambinol was used as the positive inhibitor control^15^; it was pre-incubated with human nSMase2 for 15 min prior to addition of SM. Final DMSO concentration was 0.57%. The assay exhibited signal/background = 21 and Z’ = 0.8 (Fig. 1D). We also evaluated the dose response of inhibition by cambinol and GW4869 to determine variability in the IC_50_ values from plate to plate. GW4869 was insoluble in DMSO and appeared as a yellow pellet at the 3 highest concentrations so it was excluded as a positive control. Cambinol’s average IC_50_ from 4 independent determinations was 27 ± 1 µM (Fig. 1E). The final stage of validation of the assay for HTS was the screening of the Library of Pharmacologically Active Compounds (LOPAC) in 1536-well plates using the same assay conditions at four different inhibitor concentrations (0.4, 2, 11 and 57 µM). Overall, the sample field was even, there were no plate positional effects and the number of active hits increased as the concentration increased.

### HTS campaign and data analysis of hits led to the identification of seven potent nSMase2 inhibitors

**Figure 2:**
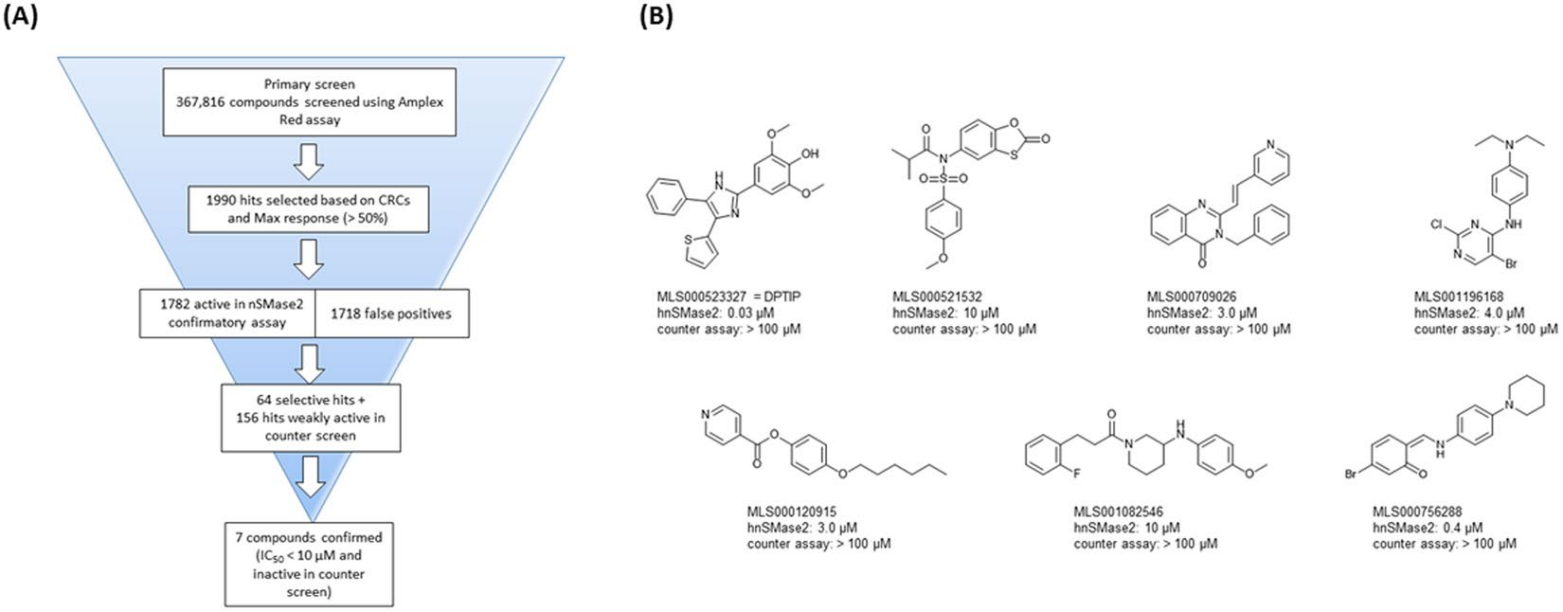
Identification of nSMase2 inhibitors from qHTS. (**A**) Flowchart illustrates the filtering of HTS hits that led to the confirmation of 7 nSMase2 inhibitors with IC_50_ < 50 µM that were inactive in the counter assay. CRC: curve response classes. (**B**) Structures of the 7 human nSMase2 inhibitors with corresponding IC_50_s for inhibition of human nSMase2. None of these compounds showed inhibition in the counter assay at 100 μM.

Following assay validation, we screened 365,000 compounds from the Molecular Libraries Small Molecule Repository (MLSMR) and 2816 compounds from the NCGC pharmaceutical collection (NPC) library for human nSMase2 inhibitors. Compounds were screened at 4 concentrations: 1.1, 11, 57 and 114 µM. Cambinol (full dose response in each plate) was used as positive control. After eliminating promiscuous compounds, 1990 compounds that had maximal inhibitory responses >50% at the highest concentration tested and robust curve response classes (CRC)^16^ were selected for re-testing in the same human nSMase2 activity assay and counter screen. The purpose of the counter screen was to identify false positives, i.e., compounds that inhibited the enzyme-coupled reactions of the assay system; it was carried out in the absence of human nSMase2 and SM and using phosphorylcholine as substrate. Out of the 1990 compounds, 1782 (90%) were confirmed in the 7 dose-response hnSMase2 confirmatory assay, but most (1718; 86%) were found to be false positives in the counter screen, resulting in 64 bona fide nSMase2 inhibitors. We also considered the difference between potency and response in the counter screen to select 156 additional hits that showed robust inhibition of the overall reaction, but were weakly active in the counter screen. There were a total of 220 compounds for follow-up confirmation (Fig. 2A). Out of the 220 compounds tested, 7 compounds exhibited dose responses with IC_50_ ≤ 10 µM that were also inactive in the counter assay (Fig. 2B).

### DPTIP is the most potent nSMase2 inhibitor reported to date

**Figure 3:**
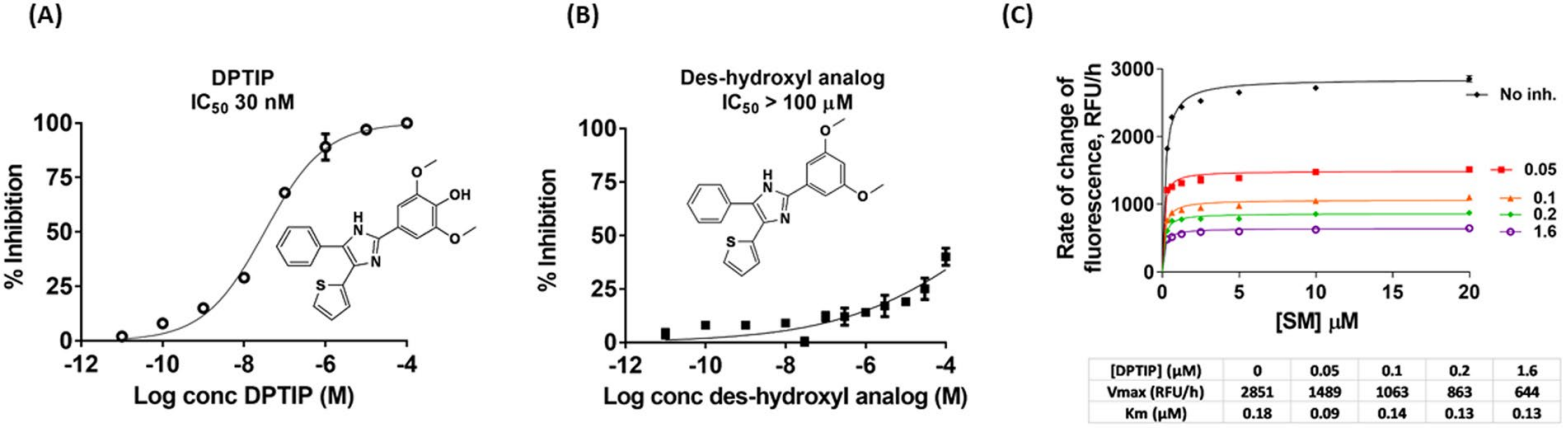
Inhibition of nSMAse2 by (**A**) DPTIP and (**B**) des-hydroxyl analog **-** Human nSMase2 (0.1 µg/µL) was added to a reaction mixture containing SM (20 µM), coupling reagents and DPTIP or JHU3398 in the 10 pM – 100 µM range. Percent inhibition was obtained from [(rate of change of fluorescence in the presence of inhibitor divided by rate of change of fluorescence in the absence of inhibitor) × 100]. Each data point corresponds to the average of two independent experiments run in replicate. Error bars correspond to S.E.M. (**C**) Rate of reaction vs concentration of SM in the presence of several DPTIP concentrations. Human nSMase2 cell lysate (0.1 µg/µL) was incubated with increasing concentrations of SM and coupling reagents for 2 h at 37 °C before measuring fluorescence. V_max_ and K_m_ values were obtained from non-linear regression fits to Michaelis-Menten kinetics using prism.

Filtering of the HTS hits as outlined above resulted in the identification of MLS000523327 or DPTIP (2,6-Dimethoxy-4-(5-Phenyl-4-Thiophen-2-yl-1H-Imidazol-2-yl)-Phenol) as the most promising compound based on potency and chemical optimization feasibility. The IC_50_ for DPTIP using an extended inhibitor concentration range (10 pM – 100 µM) was 30 nM (Fig. 3A). This IC_50_ is 30- and 160-fold more potent than the prototype inhibitors GW4869 (1 µM)^14^ and cambinol (5 µM)^15^. To our knowledge, this is the first nSMase2 inhibitor described with nanomolar potency. Because DPTIP contains a hydroxyl group which could be a metabolic liability *in vivo* (Fig. 3A), we determined the importance of this group for inhibitory activity. We synthesized the des-hydroxyl analog of DPTIP (Fig. 3B) and showed that it was inactive against human nSMase2 (IC_50_ > 100 µM) (Fig. 3B). These results demonstrate the importance of the hydroxyl group for inhibition, and also provide a structurally similar inactive DPTIP analog for use as a comparison compound in subsequent pharmacological assays.

### DPTIP exhibited non-competitive mode of inhibition and showed selectivity for nSMase2 versus related enzymes

DPTIP exhibited the hallmarks of noncompetitive inhibition; when the rate of reaction with respect to SM concentrations was monitored at increasing inhibitor concentrations, there was a decrease in maximal rate (V_max_) while the Michaelis constant (K_m_) was unchanged (Fig. 3C). V_max_ and K_m_ for each data set at a given inhibitor concentration were obtained from non-linear regression fits to Michaelis-Menten kinetics (Fig. 3C).

DPTIP did not inhibit members of two related enzyme families including alkaline phosphatase (IC_50_ > 100 µM in counter screen), a phosphomonoesterase, or acid sphingomyelinase (IC_50_ > 100 µM), a phosphodiesterase closely related to nSMase2 (results not shown). Inhibitor selectivity with respect to enzymes from related families is consistent with a noncompetitive mode of inhibition, as DPTIP is likely acting at a site other than the catalytic site. Additional data also indicate that DPTIP exhibits specificity for nSMase2; DPTIP has been screened in 759 bioassays assays at NCATS and only weak activity (2–50 µM) was observed in 19 (2.5%) of these assays. (https://pubchem.ncbi.nlm.nih.gov/compound/5446044#section=BioAssay-Results).

### DPTIP showed metabolic stability in mouse and human liver microsomes

**Figure 4:**
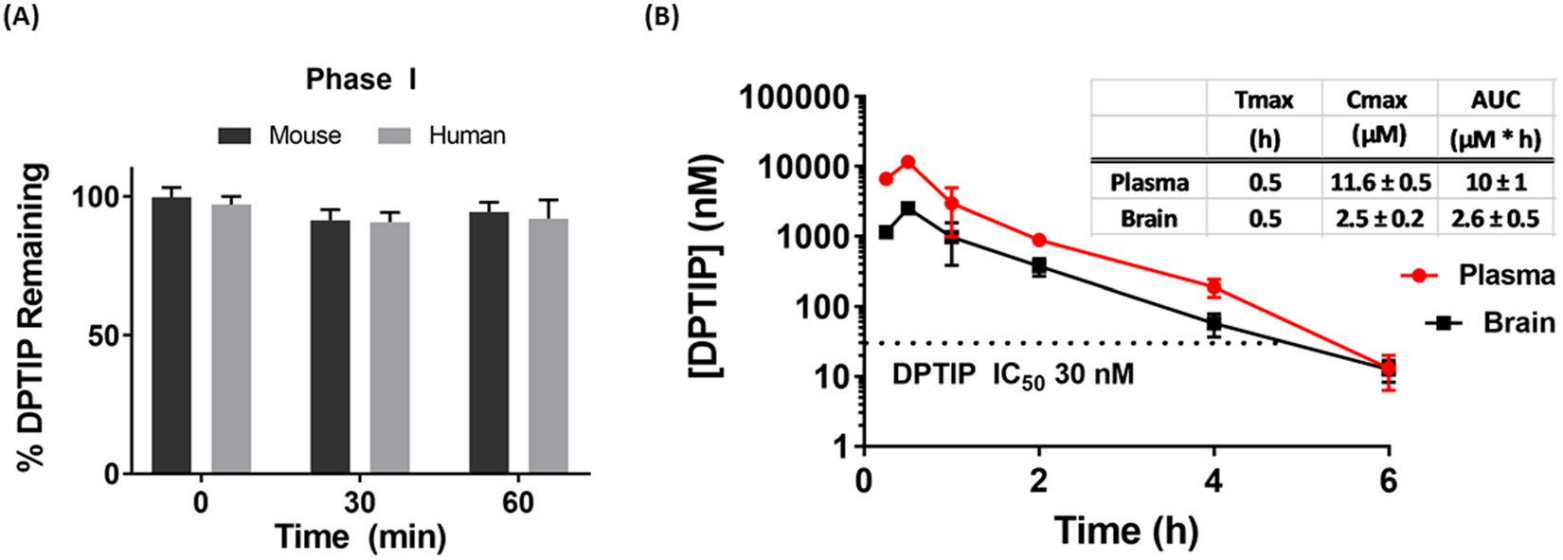
Metabolic stability and *in vivo* pharmacokinetics of DPTIP. (**A**) Metabolic stability in mouse and human liver microsomes. DPTIP was stable in mouse and human liver microsomes fortified with NADPH suggesting stability to phase I oxidation. (**B**) Plasma and brain profiles and pharmacokinetic parameters following 10 mg/kg IP dose showed DPTIP to be a brain penetrable compound with AUC_brain_/AUC_plasma_ = 0.26. Brain levels of the compound exceeded its IC_50_ for nSMase for 4 h post dose.

One potential liability when using chemical probes *in vivo* is lack of metabolic stability which structurally inactivates the compound before it can reach its molecular target. We evaluated DPTIP for metabolic stability using human and mouse liver microsomes as we have previously described^17^. Percent of drug remaining over time was determined by liquid chromatography–tandem mass spectrometry analysis (LC/MS/MS). In the presence of NADPH, DPTIP remained intact (100% remaining at 1 h) in both mouse and human liver microsomes (Fig. 4A) indicating that the compound is not affected by CYP-450-mediated metabolism. These *in vitro* results indicate DPTIP does not have major liver metabolic liabilities that would preclude its use as an *in vivo* probe.

### DPTIP exhibited plasma exposure and brain penetration after systemic dosing in mice

In the next set of experiments, we evaluated the *in vivo* pharmacokinetic profile of systemically administered DPTIP. Mice were given DPTIP (10 mg/kg IP) and plasma and brain levels of DPTIP were measured at 0.25, 0.50, 1, 2, 4 and 6 h post dose (n = 3 per time point). DPTIP peak concentration in both plasma and brain was at 0.5 h (C_max_ plasma = 11.6 ± 0.5 µM; C_max_ brain = 2.5 µM) (Fig. 4B). The AUC_0-∞_ of DPTIP in plasma and brain was 10 ± 1 and 2.6 ± 0.5 µM*h, respectively, resulting in an AUC_brain_/AUC_plasma_ = 0.26. Brain levels of DPTIP exceeded its IC_50_ for inhibition of nSMase2 up to 4 h following 10 mg/kg systemic dosing (Fig. 4B).

### DPTIP inhibited EV release from primary astrocytes whereas its inactive analog had no effect

**Figure 5:**
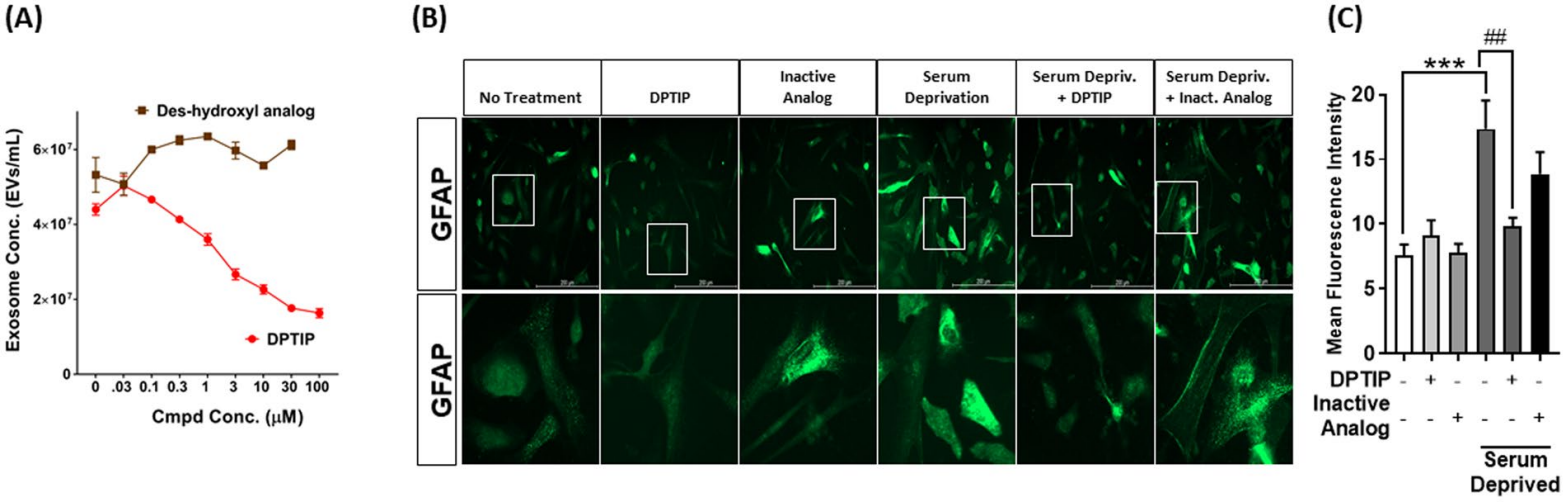
Inhibition of EV release by DPTIP in astrocytes. (**A**) Rat primary astrocytes were treated in parallel incubations with DPTIP or its des-hydroxyl inactive analog at 0.3, 1, 3, and 10 μM; DMSO (0.02%) was used as vehicle control. Media was collected after 2 h incubation and centrifuged at 2700 × g for 15 min at 4 °C. Supernatant was collected and the number of extracellular vesicles (EVs) was quantified using ZetaView Nanoparticle Tracker. The mean concentration of EVs/mL (±SEM) was calculated from 4 replicate experiments. (**B**) Rat primary astrocytes were treated with DPTIP or inactive analog (+/−) serum-deprivation-induced stress for 2 h. Astrocytes grown in complete medium were used as no treatment control. Cells were fixed and stained with anti-GFAP antibody (1:500, Sigma). Fluorescence intensity was measured using Image J. (**C**) Quantitation of fluorescence in (**B**). Bar graph represents background corrected mean fluorescence intensity measured from 100 astrocytes per condition. Error bars represent standard error of mean. One-way ANOVA followed by Tukey’s posthoc test was performed.

Independent laboratories have shown that pharmacological and genetic inhibition of nSMase2 blocks EV secretion from glial cells^12^. Consequently, we evaluated DPTIP for its ability to inhibit EV release from primary glial cells *in vitro*. Mouse primary astrocytes were activated by FBS withdrawal as we have previously described^11^ and treated with DPTIP or its closely related inactive des-hydroxyl analog (Fig. 5A) at a dose range of concentrations (0.03–30 µM) using DMSO (0.02%) as vehicle control. Two hours after treatment, EVs were isolated from the media and quantified by nanoparticle tracking analysis. DPTIP inhibited EV release from astrocytes in a dose dependent manner (Fig. 5A). In contrast, its closely related inactive analog had no effect on EV release suggesting DPTIP inhibits EV release via inhibition of nSMase2. We also determined the activation status of (+/−) serum-deprived astrocytes after DPTIP treatment. Rat primary astrocytes were treated with DPTIP (10 μM) or inactive analog for two hours along with (+/−) serum deprivation-induced stress. Cells were fixed and immunofluorescence labeling for GFAP was performed. DPTIP and inactive analog without serum starvation did not change GFAP levels (Fig. 5B,C). Serum deprivation resulted in activation of astrocytes as evidenced by increase in GFAP fluorescence intensity compared to non-treated controls. Treatment with DPTIP prevented astrocyte activation in response to serum starvation, while the inactive analog failed to prevent astrocyte activation (Fig. 5B,C).

### DPTIP inhibited biomarkers of brain inflammation *in vivo* whereas its inactive analog had no effect

**Figure 6:**
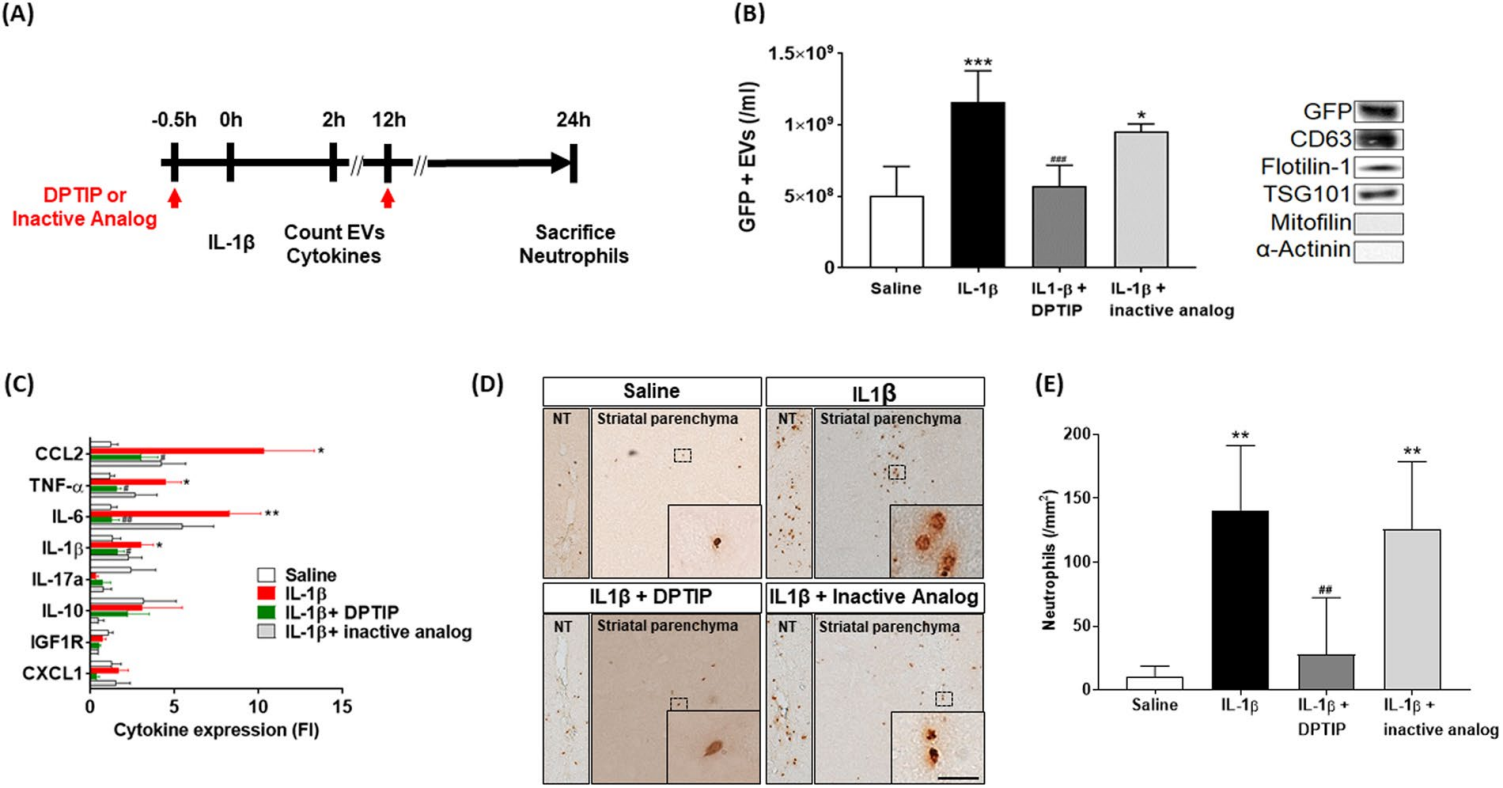
Effects of DPTIP in mouse model of brain inflammation. (**A**) Experiment Timeline – Four groups of GFAP-EGFP mice were administered saline, IL-1β, IL1-β + DPTIP (10 mg/kg) or IL-1β + inactive analog (10 mg/kg). Compounds were given 0.5 h before IL-1β dosing. One group of mice was sacrificed 2 h after IL-1β administration to determine effects of the various treatments on extracellular vesicles (EVs) releases from brain and liver cytokine analysis. The second group was dosed a second time 12 h after IL-1β administration and sacrificed at 24 h to evaluate the effects of different treatments on neutrophil infiltration into brain. (**B**) GFP-labeled EVs in plasma under different treatments. Data are mean ± SD, n = 5 mice per condition. *p < 0.05 compared to saline control; ^###^p < 0.001 compared to IL1-β group; ***p < 0.001 compared to saline group. There was no difference observed between IL-1β and IL-1β plus des-hydroxyl analog groups. Panel to the right shows Western analysis using EVs when evaluating against GFP, exosomal (CD63, flotilin-1, TSG101), mitochondrial (mitofilin) and cytoskeletal (α-actinin) markers. Full blot rows and columns are shown in Supplementary Information (Fig. 1S). (**C**) Liver cytokine levels under different treatments as measured by qRT-PCR of RNA isolated from fresh frozen liver tissue. Samples were analyzed in triplicate. **p < 0.01 and *p < 0.05 compared to saline control; ^##^p < 0.01 and ^#^p < 0.05 compared to IL1-β group. (**D**) Neutrophil levels in brain as measured by immunohistochemistry using coronal brain sections and Ly6b antibody. (**E**) Quantitation of (**D**); **p < 0.01 compared to saline control; ^##^p < 0.01 compared to IL1-β group.

Given DPTIP’s brain penetration in mice and its ability to inhibit EV release *in vitro*, we next evaluated the ability of DPTIP to ameliorate EV release from astrocytes, cytokine upregulation in liver and neutrophil migration into brain in an *in vivo* mouse model of brain inflammation. As we have previously shown^11,18^, striatal injection of IL-1β in mice expressing GFP-GFAP in astroglia triggers a release of GFP-labelled EVs that rapidly enter into plasma, resulting in cytokine upregulation in liver and peripheral immune cell migration into brain^11^. Mice were dosed (10 mg/kg IP, DPTIP or inactive analog) 0.5 h prior to IL-1β striatal injection. At this dose, brain concentrations of DPTIP are above its IC_50_ for nSMase2 inhibition for at least 4 h after compound administration (Fig. 4C). There were two groups of mice: the first group was sacrificed 2 h after IL-1β administration by heart puncture, and GFP-labeled circulating EVs were measured with liver cytokines. Mice in the second group were dosed a second time with DPTIP or inactive analog at 12 h and sacrificed at 24 h after IL-1β administration to measure brain neutrophils (Fig. 6A). Counting of astrocyte-released EV (GFP+) from blood and liver cytokine analysis was conducted by single injection of DPTIP. Although release of EVs from astrocytes can be initiated immediately after intracranial injection of IL-1β, infiltration of neutrophils in brain parenchyma occurred 12h-24h after the IL-1β injection. Since the pharmacokinetic profiles of DPTIP in plasma and brain following 10 mg/kg IP dose showed that brain levels of DPTIP exceeded its IC50 for nSMase2 for only 4 h post dose, we administered DPTIP twice after IL-1β injection to ensure inhibition of nSMase2 was sustained during the experiment. When mice were dosed with DPTIP, number of astrocyte-derived EVs was reduced by 51 ± 13% 2 h post IL-1β administration (Fig. 6B). Western analysis using the isolated exosomal fraction confirmed the presence of CD63 (transmembrane protein), TSG101 (cytosolic protein) and Flotilin-1 (lipid raft associated protein), commonly used EV markers^19,20^. The GFP signal was an indication that these EVs originated in brain^11^ while lack of mitofilin and α-actinin signals indicated the vesicles were not of mitochondrial^21^ or cytoskeletal^22^ origin respectively (Fig. 6B).

Upregulation of liver cytokines upon IL-1β treatment was inhibited by DPTIP (Fig. 6C). Neutrophils, as measured by immunohistochemistry of coronal brain sections using LY6b antibody, showed reduced staining in sections from animals treated with DPTIP compared to IL-1β-treated animals (Fig. 6D); corresponding quantification showed neutrophil migration into brain was reduced by 80 ± 23% compared to IL-1β-treated animals (Fig. 6E). Administration of the closely related inactive analog, had no statistically significant effect on IL-1β-induced EV release (Fig. 6B). The effects of the inactive des-hydroxyl DPTIP on production of TNF-α and IL-6 were marginal and not statistically significant. Although the magnitude of reduction in CCL2 production by the inactive analog was high, the data were variable and also not statistically significant (Fig. 6C). Finally, des-hydroxyl DPTIP had no effect on neutrophil migration (Fig. 6D,E). Results with the inactive analog were consistent with the suggestion that DPTIP effects occur through nSMase2 inhibition. Importantly, these results are in agreement with our previous findings that co-injection of IL-1β with nSMase2 inhibition (either GW4869, altenusin, lentivirus targeting astrocytic nSMase2, or using nSMase2 KO mice) suppress neutrophil infiltration into brain parenchyma^11^. The same studies also indicated that nSMase2 inhibition suppressed activation of astrocytes and microglia^11^.

Within this study, we focused our efforts on astrocytes because of the intimate association of these cells with the blood-brain barrier (BBB), and because in our previous study we knocked down nSMase2 expression selectively in astrocytes and showed that this inhibited the release of astrocyte-derived EVs (ADEVs) and prevented the liver cytokine response, and leukocyte trafficking into brain following parenchymal injection of IL-1beta^11^. Although it remains possible that neuronal or microglial- derived EV are also affected by nSMase2 inhibition, these earlier findings suggest that ADEVs are a major source of brain EVs that regulate the peripheral response to CNS injury. Future studies will include the use of neuronal and microglial derived EVs.

The exact mechanism of serum deprivation-induced EV release is not known. Serum deprivation is known to produce a stress response that stimulates secretory pathways in astrocytes^23^. Additionally, nutrient deprivation has been shown to cause accumulation of ceramides in astrocytes, likely due to a stress response activation of nSMase2^24^. Nutrient starvation has been reported to increase nSMase2 activity and induce its expression in other cell types^25^. Serum deprivation induced EV release observed in our experiments may therefore be the result of nSMase2 activation in response to nutrient deprivation stress.

**Figure 7:**
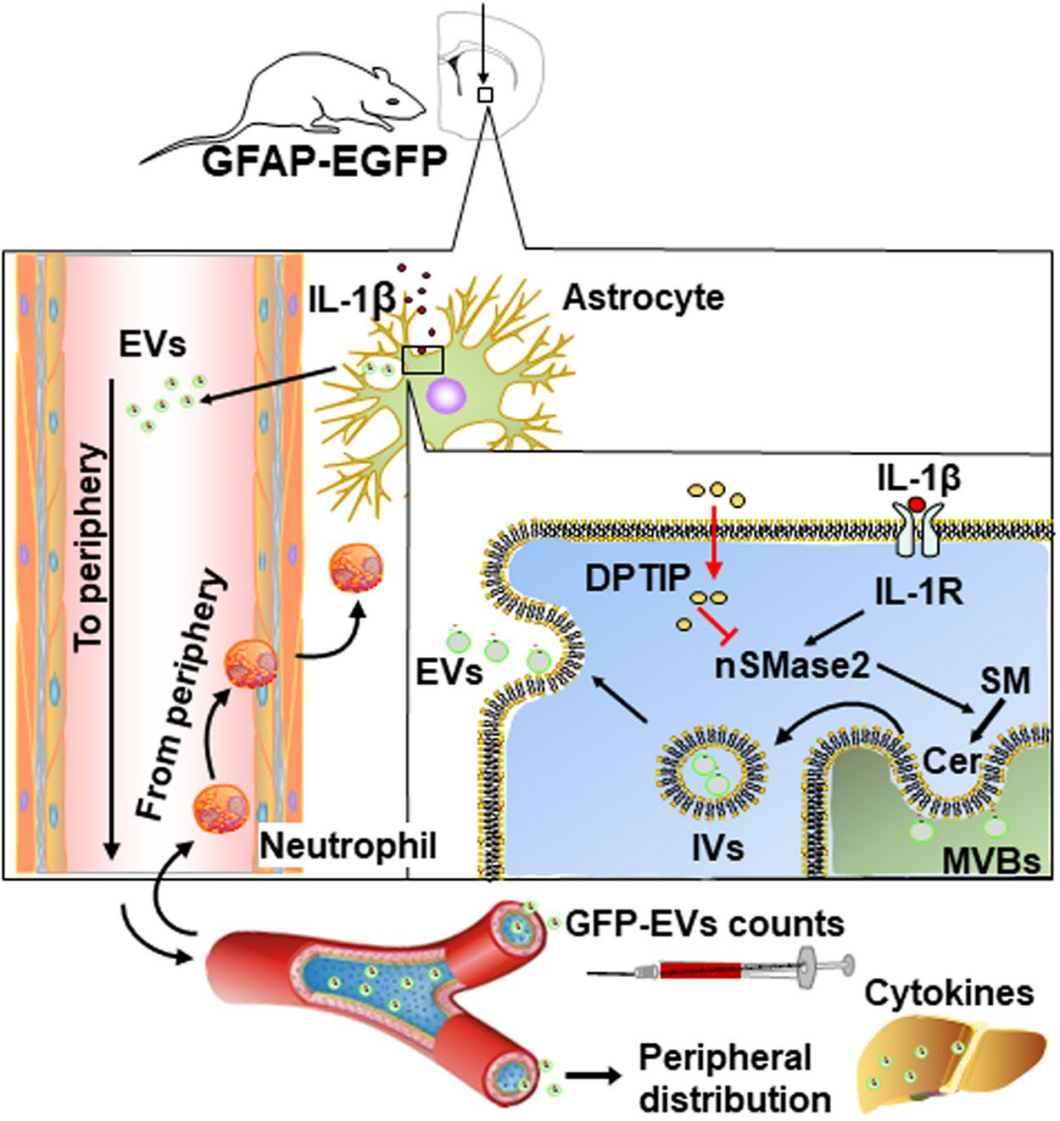
Proposed mechanism for the role of nSMase2 during inflammatory brain injury and effect of nSMase2 inhibition by DPTIP – Intracerebral injection of IL-1β activates the IL-1β receptor which in turn activates nSMase2. nSMase2-catalyzed hydrolysis of sphingomyelin (SM) produces long-chain ceramides (Cer). Increase in ceramide production at multivesicular bodies (MVBs) leads to the formation of intraluminal vesicles (IVs) and budding of extracellular vesicles (EVs) that are then shed from astrocytes and released into the periphery. Astrocyte-generated EVs can be identified in plasma because they are GFP-labeled. Astrocytic EVs promote crossing of neutrophils into brain as a result of cytokine upregulation in liver. In the presence of DPTIP, nSMase2 is inhibited, ceramide is not available for EV biosynthesis resulting in blockade of both cytokine upregulation and neutrophil infiltration.

A schematic illustration of the *in vivo* experiment are shown in Fig. 7 which are consistent with the data detailed above as well as previous literature. In brief, striatal IL-1β injection activates the IL-1β receptor on the plasma membrane of astrocytes that in turn activates nSMase2 enzymatic activity to catalyze the hydrolysis of sphingomyelin to produce ceramide^26^. Ceramide is used to manufacture intracellular vesicles (IVs)^1^ that are released from astrocytes as EVs and migrate into plasma where they induce a peripheral acute cytokine response, mainly in liver, and prime immune cells to transmigrate to the brain^11^. In the presence of DPTIP, inhibition of nSMase2 prevents ceramide production, EV formation and secretion (Fig. 6B) cytokine upregulation (Fig. 6C) and neutrophil migration (Fig. 6D).

In summary, DPTIP is the most potent nSMase2 inhibitor identified to date (IC_50_ 30 nM), exhibits selectivity, is metabolically stable and brain penetrant. DPTIP is an inhibitor of EV release in primary glial cells and *in vivo*. In addition, biomarkers that have been associated with EV release from brain, including cytokine upregulation and immune cell migration to brain, were also inhibited by DPTIP. The des hydroxyl inactive analog of DPTIP did not inhibit EV release *in vitro* and had no effect on IL-1β-induced cytokine regulation or neutrophil migration to brain *in vivo*. DPTIP is a considerable improvement over other nSMase2 inhibitors identified to date, it can be used as a probe in animal models of disease associated with EV dysregulation and it contains a structural scaffold that is actively being optimized for clinical translation.

## Methods

### Expression of human nSMase2

Full length human nSMase2 cDNA with a C-terminal Flag tag cloned into a pCMV6-Entry expression vector (Origene) was transfected into HEK293 cells using lipofectamine 2000 (Life Technologies). Selection of transfected cells was carried out for two weeks with 500 µg/ml G418 in EMEM containing 10% FBS (ATCC) and 2 mM glutamine (Life Technologies). Expression of human nSMase2 was confirmed by Western-blot analysis using an antibody specific against nSMase2 (R&D) diluted to 0.4 µg/ml in Tris-buffered saline with 0.1% Tween 20 and 5% bovine serum albumin. Cells expressing human nSMase2 were grown to confluency in 150 mm dishes, washed twice with cold PBS and harvested using a cell scraper in lysis buffer pH 7.5, Tris-HCl 100 mM, 1 mM EDTA, 100 mM sucrose, 100 µM PMSF, 1X protease inhibitor cocktail III (Calbiochem), 1 ml per dish. Cell lysis was achieved by sonicating 3 times on ice for 30 sec. Protein concentration was determined using the bicinchoninic acid (BCA) assay. Aliquots of cell lysate were snap frozen and stored at −80 °C. Activity of recombinant human nSMase2 from cell lysates remained stable for at least six months.

### Fluorescence-based nSMase2 activity assay in 1536-well format

Measurements of nSMase2 activity using fluorescence as readout was optimized for dose response quantitative HTS (qHTS). The assay was carried out in black solid bottom, medium binding, 1536-well plates (Greiner, 789176-F). Fluorescence response was optimized with respect to nSMase2 concentration, incubation time and SM concentration. Recombinant human nSMase2 preparations (2 µL) at various concentrations (0.03 to 0.5 µg protein/µL solution) were incubated with substrate/detection reaction mixture (2 µL) containing various concentrations of SM (5 to 40 μM), coupling enzymes (alkaline phosphatase 4 U/mL, choline oxidase 0.1 U/mL and horseradish peroxidase 0.1 U/mL) and Amplex red® (50 μM). Hydrolysis of SM was carried out for different incubation times (15–160 min) at 37 °C in pH 7.4 Tris-HCl buffer 100 mM, containing 10 mM MgCl_2_ and 0.2% Triton X-100. Phosphorylcholine made during the nSMase2-catalyzed reaction is dephosphorylated by alkaline phosphatase to produce choline, which in turn undergoes oxidation in the presence of choline oxidase to produce betaine and peroxide. Peroxide in the presence of horseradish peroxidase and Amplex Red generates fluorescent resorufin (Ex 525/Em 598). Resorufin was monitored with Viewlux µHTS Microplate Imagers (Perkin Elmer) at energy levels 1,000 or 3,000 and exposure times of 1 or 2 sec. Fluorescence readings varied when using Viewlux offline (assay characterization) vs. Viewlux online (HTS); in order to account for differences in fluorescence efficiency, assay performance was monitored from machine to machine based on assay dynamic range and cambinol IC_50_ reproducibility. Based on results of the different conditions outlined above, the HTS campaign was carried out using 0.1 µg protein/µL nSMase2 preparation, 20 μM SM and 2 h time of incubation. Control inhibitors or test compounds (23 nL) were added from various concentrations in DMSO solution into to the nSMase2 preparation and incubated for 15 min prior to the addition of substrate and enzyme-coupling detection reagents. Compounds were screened in 4 doses, starting at 57 µM, and doing 5-fold dilutions. A customized screening robot (Kalypsys) was used for the primary screen. A step-by step HTS assay protocol is given in the Supplementary Data (Table S1). Inhibitors of nSMase2 were selected using compound dose response curve algorithms developed at NCGC to score actives, which assigns each tested compound a compound response class (CRC) number^16^. This method classifies primary hits into different categories according to their potency (IC_50_), magnitude of response (efficacy), quality of curve fitting (r2), and number of asymptotes. For example, CRC of −1.1 represents complete curve and high efficacy; CRC of −1.2 represents complete curve but partial efficacy. Compounds with CRCs of −1.1, −1.2, −2.1 and −2.2 were generally selected for confirmation and validation. Structural analysis of selected compounds was performed and promiscuous compounds were filtered out. A counter-assay to rule out compounds that inhibited the detection reaction was carried out in the absence of human nSMase2. The reaction was initiated with addition of phosphorylcholine (alkaline phosphatase substrate), added at a final concentration of 2 μM. Compounds that showed inhibitory activity in the counter-assay were removed from further validation.

### IC_50_ determination of selected compounds

Human nSMase2 (0.1 µg protein/µL) was added to a reaction mixture containing SM (20 µM), and detection reagents as indicated above and different compound concentrations in the 10 pM – 100 µM range in a total volume of 100 µL (96-well format). Kinetic measurements were obtained from 2 h traces when the reaction was linear. Percent inhibition was obtained using the formula [(rate of change of fluorescence in the presence of inhibitor divided by rate of change of fluorescence in the absence of inhibitor) × 100].

### Synthesis and characterization of DPTIP and des-hydroxyl DPTIP

Detailed descriptions of the synthesis of DPTIP and its des-hydroxyl analog along with corresponding authentication information are given in Supplementary Data.

### Metabolic stability

Metabolic stability assay was conducted in mouse or human liver microsomes as we have described previously with minor modifications^17^. Briefly, the reaction was carried out using potassium phosphate buffer (100 mM, pH 7.4), in the presence of an NADPH regenerating system (compound final concentration was 1 μM; 0.2 mg/mL microsomes). Compound disappearance was monitored over time using a liquid chromatography and tandem mass spectrometry (LC/MS/MS) method. Chromatographic analysis was performed using an Accela ultra high-performance system consisting of an analytical pump and an autosampler coupled with a TSQ Vantage mass spectrometer (Thermo Fisher Scientific Inc., Waltham, MA). Separation of analyte was achieved at ambient temperature using Agilent Eclipse Plus column (100 × 2.1 mm i.d.) packed with a 1.8 μm C18 stationary phase. The mobile phase consisted of 0.1% formic acid in acetonitrile and 0.1% formic acid in water with gradient elution. The [M + H]^+^ ion transition of DPTIP (m/z 378.956 → 363.073, 200.055) and losartan (IS) (m/z 423.200 → 207.107, 180.880).

### *In vivo* pharmacokinetics

Pharmacokinetic studies in mice were approved by the Animal Care and Use Committee at Johns Hopkins University. Male CD1 mice between 25 and 30 g were obtained from Harlan and maintained on a 12 h light−dark cycle with ad libitum access to food and water. Test compounds were dosed at 10 mg/kg IP at a dosing volume of 10 mL/kg. Blood and brain tissue were collected at 0.25, 0.5, 1, 2, 4 and 6 h post dose (n = 3 per time point). Blood was obtained via cardiac puncture and plasma was harvested from blood by centrifugation at 3000 × g for 15 min and stored at −80 °C. Brain tissues were harvested following blood collection and immediately snap frozen in liquid nitrogen and stored at −80 °C until LC−MS analysis. Calibration standards were prepared using naïve mouse plasma or brain spiked with DPTIP. DPTIP standards and samples were extracted from plasma and brain by a one-step protein precipitation using acetonitrile (100% v/v) containing internal standard (losartan: 0.5 µM). The samples were vortex mixed for 30 secs and centrifuged at 10000 × g for 10 min at 4 °C. Fifty microliter of the supernatant was diluted with 50 µL water and transferred to a 250 µL polypropylene vial sealed with a Teflon cap and analyzed via LC/MS/MS as described above. Plasma concentrations (pmol/mL) as well as tissue concentrations (pmol/g) were determined and plots of mean plasma concentration versus time were constructed for PK analysis. Non-compartmental-analysis modules in Phoenix WinNonlin version 7.0 (Certara USA, Inc., Princeton, NJ) were used to assess pharmacokinetic parameters including maximal concentration (C_max_), time to C_max_ (T_max_), and area under the curve extrapolated to infinity (AUC_0-∞_).

### Inhibition of EV release from primary glial cells

Potential inhibition of test compounds on EV release from primary astrocytes was carried out as previously described (Dickens *et al*., 2017). Briefly, rat primary astrocytes were seeded onto 6-well plates at a density of 20,000 cells/well. Twenty-four hours after seeding, astrocytes were washed with PBS and the medium changed to media without FBS. Absence of FBS mimics a trophic factor withdrawal stimulus causing EVs to be released from astrocytes via an nSMase2-dependent pathway. Astrocytes were then treated with test compounds at different concentrations: 0.03, 0.1, 0.3, 1, 3, and 10 μM. DMSO (0.02%) was used as control. Two hours after treatment, media was collected and centrifuged at 2700 × g for 15 min at 4 °C. The supernatant was collected and the number of EVs quantified using ZetaView Nanoparticle Tracker (Particle Metrix GmBH, Meerbusch, Germany) and the corresponding ZetaVeiw software (8.03.04.01). Nanosphere size standard 100 nm (Thermo Scientific) was used to calibrate the instrument prior to sample readings. Instrument pre-acquisition parameters were set to 23 °C, a sensitivity of 65, a frame rate of 30 frames per second (fps), a shutter speed of 100, and laser pulse duration equal to that of shutter duration. Post-acquisition parameters were set to a minimum brightness of 25, a maximum size of 200 pixels, and a minimum size of 10 pixels. For each sample 1 mL of the supernatant was injected into the sample-carrier cell and the particle count measured at 5 positions, with 2 cycles of reading per position. The cell was washed with PBS after every sample. Mean concentration of EVs/mL (±SEM) was calculated from 4 replicates.

### Inhibition of EV release *in vivo*

All experimental protocols using vertebrate animals were reviewed by the Institutional Animal Care and Use Committee at Johns Hopkins University and are in accordance with the guidelines of the NIH guide for the care and use of laboratory animals. Striatal injections and EV measurements were performed as previously described by our group in adult (2–3 month) male GFAP-GFP mice (Jackson Laboratories)^11,18^. Mice were anesthetized with 3% Isoflourane (Baxter) in oxygen (Airgas), and placed in a stereotaxic frame (Stoelting Co.). A small burr hole was drilled in the skull over the left striatum using a dental drill (Fine Scientific Tools). IL-1β (0.1 ng/3 µL) was injected (total volume of 3 μL) at the rate 0.5 µL/min *via* a pulled glass capillary tip diameter <50 µm^18^; using the stereotaxic coordinates: A/P + 0.5; M/L −2; −3 D/V. Saline was used as a control. When DPTIP or its des-hydroxyl analog were used, they were given IP (10 mg/kg, 5% DMSO, 5% Tween-80 in saline) 30 min before IL-1β injection. Following infusion, the capillary was held in place for 5 min to allow for solution to diffuse into the tissue. Animals were sacrificed at 2 h by an overdose of anesthetic, and transcardially perfused with ice-cold saline containing heparin (20 µL per 100 ml, Sigma). Blood was collected *via* cardiac puncture using a heparin (Sigma Aldrich) coated syringe and EDTA tubes (BD) 2 h following striatal injections. Blood was immediately centrifuged at 2,700 x *g* for 15 min (20 °C) to obtain plasma. Plasma was further centrifuged at 10,000 g for 15 min (4 °C) to generate platelet free plasma. This procedure removes large particles such as apoptotic bodies.

#### Quantitation of *Plasma EVs*

Dynabeads M-450 Epoxy (Invitrogen) were coupled with anti-GFP antibody (Thermo Fisher) at a ratio of 200 μg antibody per 4 × 10^8^ beads. Plasma from GFAP-GFP mouse (50 μL) was incubated with 2 × 10^7^ anti-GFP antibody-coupled Dynabeads at 4 °C overnight. The beads were washed and placed on a magnet to separate EVs bound to anti-GFP antibody-coupled Dynabeads. The precipitated EVs were eluted using 0.1 M glycine (pH 3.0). The concentration of immunoprecipitated GFP + EVs was quantified using ZetaView nanoparticle tracking analysis (Particle Metrix) as described above.

#### Western analysis

Proteins were resolved by 10% SDS–polyacrylamide gel electrophoresis and transferred to polyvinylidene difluoride membranes (Bio-Rad). Nonspecific binding sites were blocked with 5% (w/v) milk in TBS containing 0.1% Tween 20 (TBS-T). After blocking, blots were incubated overnight with the primary polyclonal antibodies to GFP (1:1000; Thermo Fisher) CD63 (1:200; Santa Cruz Biotechnology), flotillin1 (1:1000; Abcam), TSG101 (1:1000; BD Biosciences), mitofilin (1:5000 Thermo Fisher Scientific) and α-actinin (1:1000; Gentex). After washes with TBS-T, blots were incubated for 2 h with the corresponding IgG horseradish peroxidase–linked secondary antibody (1:1000; Cell Signaling Technology) and developed by enhanced chemiluminescence. Image analysis was performed using a G: BOX imaging system (Syngene).

#### Cytokine measurements

RNA was isolated from fresh frozen tissues (10 to 50 mg) using the RNeasy Mini Kit (Qiagen). Total RNA was reverse-transcribed and quantified using previously published methods^27^. For quantitative real-time PCR (qRT-PCR), each reaction contained SYBR Green Master Mix (12.5 ml; Life Technologies), diethyl pyrocarbonate H_2_O (10.5 ml), forward and reverse primers to CCL2, TNFα, IL-6, IL-1b, IL-17a, IL-10, IGFR1, and CXCL1 (0.5 ml each; Sigma-Aldrich), and cDNA (1 ml). Each 96-well plate included a nontemplate control, and samples were analyzed in triplicate on an Applied Biosystems 7300 (Life Technologies). Cycling parameters were as follows: one cycle for 2 min at 50 °C, one cycle for 10 min at 95 °C, and 40 cycles for 15 s at 95 °C and for 1 min at 60 °C. The change in threshold cycle (ΔC_t_) for each sample was normalized to β-actin, and ΔΔC_t_ was calculated by comparing ΔC_t_ for the treatment group to the average ΔC_t_ of the control group^28^.

#### Immunohistochemistry

Coronal brain sections (30 µm) were prepared using a cryostat microtome (Leica). Endogenous peroxidase activity was quenched using a 1% solution of H_2_O_2_ in methanol, and primary antibody Ly6b (1:1000, AbD Serotec), was incubated at 4 °C overnight. Sections were washed (3 × PBS), and biotinylated secondary antibody (1:100, Vector Laboratories) was added at room temperature for 2 hours. Staining was visualized using an avidin-biotin complex (1:100 of A and B, Vector Laboratories) and DAB-HCl using a microscope to monitor staining progression. Stereological quantitation was performed using a one-in-five series (270-µm spacing), from the rostral point of bregma +1.10 mm to the caudal point of bregma −0.58 mm as previously described^29^.

### Ethical approval

All experimental protocols using vertebrate animals were reviewed by the Institutional Animal Care and Use Committee at Johns Hopkins University and are in accordance with the guidelines of the NIH guide for the care and use of laboratory animals. Johns Hopkins Medical Institution is fully accredited by the American Association for Accreditation in Laboratory Animal Care (AAALAC).

## Electronic supplementary material

Supplementary Information

## Data Availability

Experimental data used to generate the results reported in this manuscript are available upon request.
