# Supplementary material for: DPTIP, a newly identified potent brain penetrant neutral sphingomyelinase 2 inhibitor, regulates astrocyte-peripheral immune communication following brain inflammation
Source: Sci Rep. 2018 Dec 7;8:17715. doi: 10.1038/s41598-018-36144-2 (PMC6286365; doi:10.1038/s41598-018-36144-2)

SUPPLEMENTARY INFORMATION

DPTIP, a newly identified potent brain penetrant neutral sphingomyelinase 2 inhibitor, regulates astrocyte-peripheral immune communication following brain inflammation

Camilo Rojas[1,2], Elena Barnaeva[7], Ajit G. Thomas[1], Xin Hu[7], Noel Southall[7], Juan Marugan[7], Amrita Datta Chaudhuri[3], Seung-Wan Yoo[3], Niyada Hin[1], Ondrej Stepanek[1], Ying Wu[1], Sarah C. Zimmermann[1,3], Alexandra G. Gadiano[1], Takashi Tsukamoto[1,3], Rana Rais[1,3], Norman Haughey[3*], Marc Ferrer[7*], Barbara S. Slusher[1,3,4,5,6,8 *]*

[1]Johns Hopkins Drug Discovery, Departments of [2]Molecular and Comparative Pathobiology, [3]Neurology, [4]Psychiatry and Behavioral Sciences, [5]Neuroscience, [6]Medicine, [8]Oncology, Johns Hopkins School of Medicine, Baltimore, Maryland 21205, U.S.A.

[7]National Center for Advancing Translational Sciences (NCATS), National Institute of Health, Bethesda, MD 20892-3370

*** Corresponding authors**
crojas2@jhmi.edu
nhaughe1@jhmi.edu
marc.ferrer@nih.gov
bslusher@jhmi.edu

Table S1 – Detailed assay protocol to screen for human nSMase2 inhibitors

Step	Parameter	Value	Description
1	Enzyme: hSMase2 in cell lysate	2 µL /well	0.2 µg protein/µL as 2x working concentration in 1x reaction buffer on black, medium binding, solid bottom 1536-well plate (Greiner 789176-F): columns (C) 4-48
2	(-) control	2 µL /well	C2: no enzyme
3	(+) control	2 µL /well	C3: Bacterial enzyme at 0.04 U/ml as 2x working concentration
4	Centrifugation	1000 rpm, 20 sec	
5	Control Inhibitor	23 nL	C1: Cambinol 50 mM, dose response 1:2 dilutions
6	Pre-incubation time	15 min	Ambient temperature
7	Reaction mix including substrate (SM)	2 µL /well	Amplex Red + HRP + choline oxidase + alkaline phosphatase mix containing 0.04 mM sphingomyelin as 2X working concentration to all wells
8	Centrifugation	1000 rpm, 20 sec	
9	Incubation time	120 min	37 °C
10	Detection	Viewlux	Fluorescent settings: Excitation 525 / Emission 598, Energy 3000, Exposure 2 sec

Step	Notes
1	Keep enzyme on ice
4	Centrifugations in steps 4 & 9 are carried out to insure reagents are mixed and at the well's bottom
7	Keep substrate mix on ice and protect bottle and tubing from light. Keep room light dimmed.
9	Pre-read is carried out to exclude fluorescent compounds.

Synthesis and authentication of DPTIP

The synthesis of DPTIP is outlined in the scheme below:

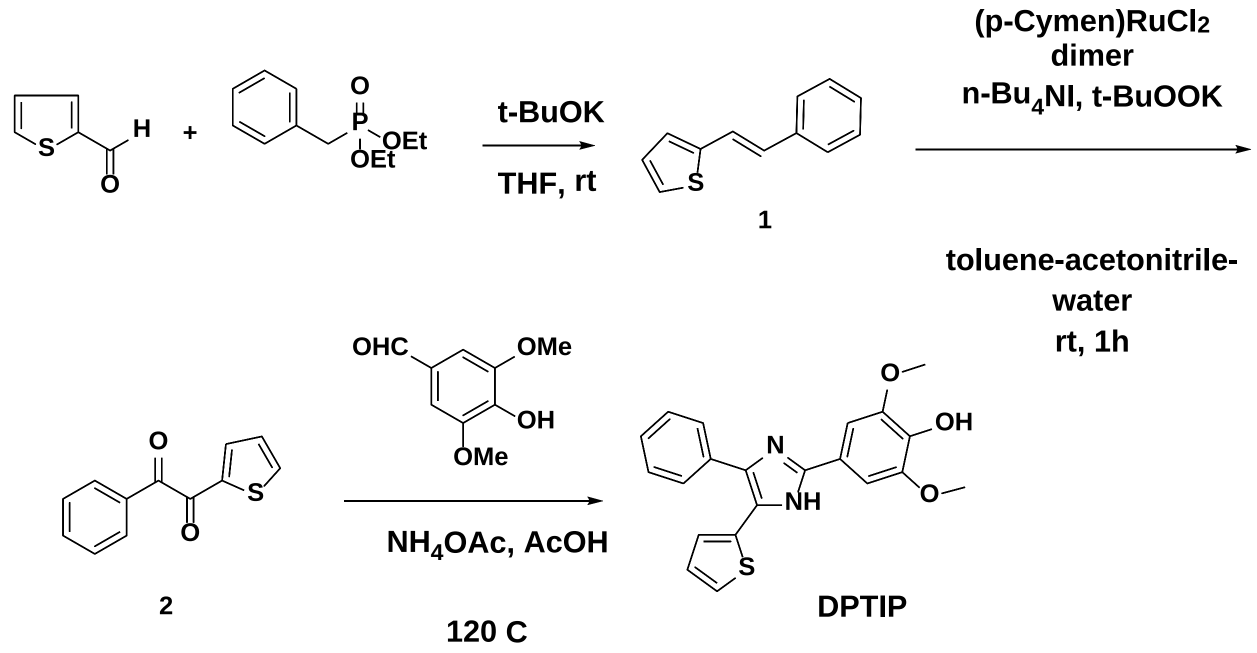

*Synthesis of compound **1** (2-styrylthiophene)*: To a cooled solution mixture of diethyl benzylphosphonate (13.3 g, 58.5 mmol) and thiophene-2-carbaldehyde (6.56 g, 58.5 mmol, 1 equiv) at 0 °C in THF (25 mL) was added a solution of t-BuOK (12.5 g, 111.1 mmol, 1.9 equiv) in THF (80 mL) via addition funnel. At the end of the addition, the guey mixture was stirred at 0 °C, then gradually allowed to warm to rt overnight. Ethyl acetate was added. The organic layer was washed with water and brine, dried over sodium sulfate and concentrated. Trituration of the crude material in 15% EtOAc/hexanes gave 6.06 g (56%) of 2-styrylthiophene (**1**) as a beige solid. ^{1}H NMR (400 MHz, $CDCl_3$) δ 7.46 (d, *J*= 7.4 Hz, 2H), 7.36 (t, *J*= 7.6 Hz, 2H), 7.28 (m, 1H), 7.20 –7.23(m, 2H), 7.07 (d, *J*= 3.2 Hz, 1H), 7.01 –7.03 (m, 1H), 6.92 (d, *J*= 16.4 Hz, 1H).

*Synthesis of compound **2** (1-phenyl-2-(thiophen-2-yl)ethane-1,2-dione)*: 2-Styrylthiophene (**1**, 6.06 g, 32.5 mmol), dichloro(*p*-cymene) ruthenium(II) dimer (0.20 g, 0.33 mmol, 0.01 equiv) and n-Bu_4NI (3.60 g,

9.76 mmol, 0.3 equiv) were combined together in a flask. Toluene (100 mL) and acetonitrile (100 mL) were added, followed by water (50 mL). *Tert*-butyl hydroperoxide (42 mL) was then slowly added via addition funnel at 0 °C. The reaction mixture was stirred at 0 °C and gradually allowed to warm up and stirred at rt for 1 h then quenched with saturated aqueous Na_2SO_3 solution. The product was extracted with EtOAc (x2). The organic layer was washed with brine and dried over sodium sulfate. Purification by Biotage (120 g silica column, 5-10% EtOAc/hexanes) gave 5.03 g (71%) of 1-phenyl-2-(thiophen-2-yl)ethane-1,2-dione (**2**) as a yellow oil which solidified to a yellow solid upon drying. ^{1}H NMR (400 MHz, CDCl3): δ 8.05 (dd, *J* = 1.5, 8.6 Hz, 2H), 7.85 (dd, *J* = 1.0, 4.8 Hz, 1H), 7.80 (dd, *J* = 1.3, 4.0 Hz, 1H), 7.66-7.70 (m, 1H), 7.53 (m, 2H), 7.19-7.22 (dd, *J* = 3.8, 4.8 Hz, 1H).

Synthesis of DPTIP: Diketone **2** (0.5 g, 2.31 mmol), 4-hydroxy-3,5-dimethoxybenzaldehyde (0.46 g, 2.54 mmol, 1.1 equiv) and ammonium acetate (1.78 g, 23.1 mmol, 10 equiv) were heated together in acetic acid (15 mL) at 120 °C overnight. The next day, the reaction was concentrated in vacuo. The crude material was extracted with EtOAc and water. The organic layer was washed with brine, dried over sodium sulfate and concentrated. The crude material was triturated in 20% EtOAc/hexanes (with a small amount of methanol) to give 0.73 g (83%) of DPTIP as a dark purple solid.

Authentication - The chemical structure was characterized by ^{1}H NMR data, HPLC, mass spectrometry, and melting point.

- ^{1}H NMR (400 MHz, d6-DMSO): δ 3.84 (6H, s), 6.96 (1H, dd, *J* = 3.8 Hz, 5.1 Hz), 7.03 (1H, dd, *J* = 1.3 Hz, 3.8 Hz), 7.32 (2H, s), 7.37 (1H, m), 7.43 (1H, m), 7.50 (2H, t, *J* = 7.8 Hz), 7.58 (2H, d, J = 7.1 Hz), 8.67 (1H, s), 12.52 (1H, s);
- ^{13}C NMR (100 MHz, d6-DMSO): δ 148.1, 146.0, 138.4, 136.2, 131.6, 130.7, 128.8, 128.7, 128.1, 127.3, 127.1, 124.1, 122.4, 120.4, 102.8, 56.1; Anal. Calcd. For $C_{21}H_{18}N_2O_3S$.0.15 Formic acid: C, 65.92; H, 4.79; N, 7.27; S, 8.32. Found: C, 65.83; H, 4.74; N, 7.32; S, 8.26.
- M.P. > 250 °C;

- MS calculated m/z for $C_{21}H_{18}N_2O_3S$: 378; $[M+H]^+$ found: 379.

- HPLC chromatogram of purified product showed 1 single peak with retention time at 1.5 min

Synthesis and authentication of des-hydroxyl analog of DPTIP

The synthesis of the des hydroxyl analog of DPTIP is outlined in the scheme below.

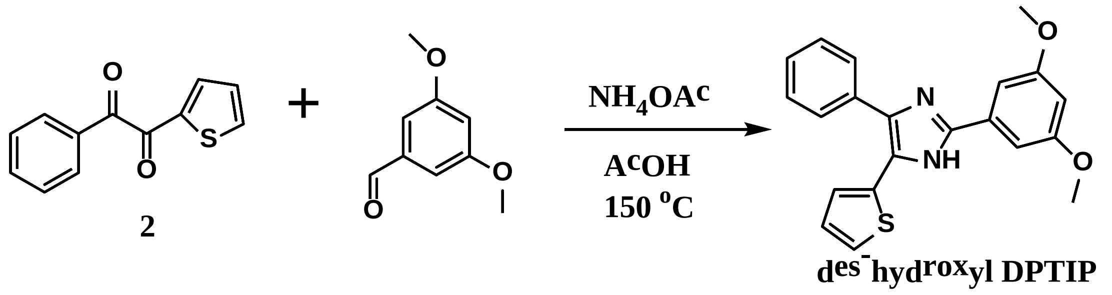

Diketone **2** (60 mg, 0.28 mmol), 3,5-dimethoxybenzaldehyde (55 mg, 0.33 mmol, 1.2 equiv) and ammonium acetate (214 mg, 2.77 mmol, 10 equiv) were heated together in acetic acid (3 mL) at 150 °C overnight. The next day, the reaction was concentrated in vacuo. The crude material was extracted with EtOAc and water. The organic layer was washed with brine, dried over sodium sulfate and concentrated. The resulting material was purified by Biotage (eluent: 20-40% EtOAc/hexanes) to give 75 mg (75% yield) of 2-(3,5-dimethoxyphenyl)-4-phenyl-5-(thiophen-2-yl)-1H-imidazole (des-hydroxyl DPTIP) as a white solid.

Authentication - The chemical structure of des-hydroxyl DPTIP was characterized by ^{1}H NMR data, HPLC, mass spectrometry, and melting point.

- ^{1}H NMR (400 MHz, d6-DMSO): δ 3.81 (6H, s), 6.51 (1H, t, J = 2.1 Hz), 6.97 (1H, dd, J = 3.8 Hz, 5.1 Hz), 7.04 (1H, dd, J = 1.3 Hz, 3.5 Hz), 7.23 (1H, d, J = 2.3 Hz), 7.34 (1H, s), 7.37 (1H, dd, J = 1.3 Hz, 5.1 Hz), 7.44 (1H, m), 7.51 (2H, t, J = 7.3 Hz), 7.59-61 (2H, m), 12.74 (1H, s).

- ^{13}C NMR (100 MHz, d6-DMSO): 160.7, 145.2, 138.2, 132.0, 131.8, 130.5, 128.9, 128.7, 128.3, 127.8, 127.4, 124.3, 122.6, 103.1, 100.7, 55.4.

- M.P = 239-241 °C
- MS - calculated m/z for $C_{21}H_{18}N_2O_2S$: 362; $[M+H]^+$ found: 363.
- HPLC chromatogram of purified product showed one single peak at 1.85 min.

Fig 1S - Blots corresponding to cropped blot in Fig 6b –

Cropped column shown below was used for Fig 6b

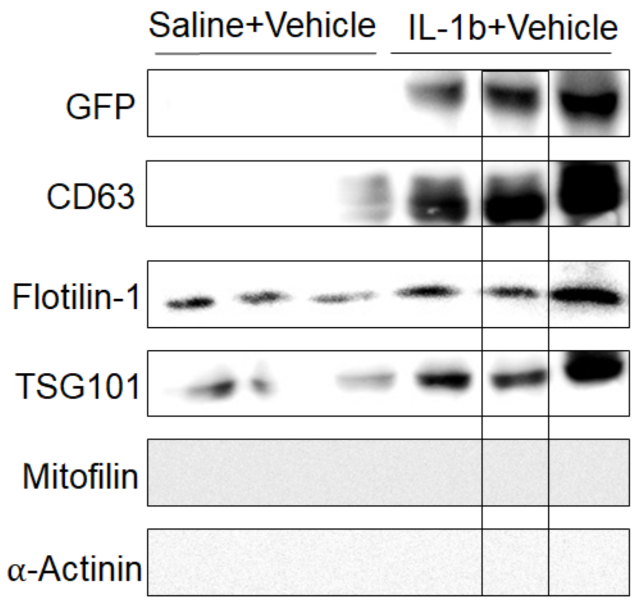

Supplement: Supplementary file 1 — Supplementary Information [file 41598_2018_36144_MOESM1_ESM.pdf]
